# Qualitative and Quantitative Correlation of Microstructural Properties and In Vitro Glucose Adsorption and Diffusion Behaviors of Pea Insoluble Dietary Fiber Induced by Ultrafine Grinding

**DOI:** 10.3390/foods11182814

**Published:** 2022-09-13

**Authors:** Lingyi Li, Jianfu Liu, Yang Zhang, Qian Wang, Jinrong Wang

**Affiliations:** 1 Tianjin Key Laboratory of Food and Biotechnology, School of Biotechnology and Food Science, Tianjin University of Commerce, No. 409 Guangrong Road, Beichen District, Tianjin 300134, China; 2 Tianjin International Joint Research and Development Center, No. 409 Guangrong Road, Beichen District, Tianjin 300134, China

**Keywords:** dietary fiber, ultrafine grinding, microstructure, glucose adsorption and diffusion, quantitative correlation

## Abstract

Ultrafine grinding is an important pretreatment to achieve the physical modification of dietary fiber. In this study, ultrafine grinding treatments were performed for different times to give pea insoluble dietary fiber (PIDF) samples with varied particle sizes (*D*_50_). The correlations and quantitative relationships between the microstructures of multi-scales PIDF and its in vitro glucose adsorption and diffusion behaviors were comprehensively evaluated. The results indicated that the specific surface area (*SSA*), pore volume (*PV*) and oxygen-to-carbon surface ratio (*O/C*) of PIDF were significantly increased by ultrafine grinding at the cellular scale, while *D*_50_ and cellulose crystallinity (*CrI*) were significantly decreased. These changes significantly improved the glucose adsorption capacity (*GAC*) of PIDF. The order of importance of microstructural changes on *GAC* was *O/C* > *PV* > *SSA* > *CrI* > *D*_50_. *GAC* showed positive exponential relationships with *SSA*, *PV*, and *O/C* and showed a negative linear relationship with *CrI*. The ability to retard glucose diffusion increased significantly with decreased fiber particle size because of improved adsorption and interception of glucose and the dense physical barrier effect of PIDF. The quantitative equation of maximum glucose dialysis retardation index was *GDRI*_max_ = −1.65 ln(*D*_50_) + 16.82 ln(*GAC*) − 68.22 (*R*^2^ = 0.99). The results could provide theoretical support for quantitative and targeted intervention of dietary fiber structure for blood glucose control.

## 1. Introduction

Pea is one of the main edible legumes that is widely planted throughout the world. As a by-product from the processing of peas, pea seed coats are mostly used as animal feed or are discarded, resulting in serious environmental pollution and resource waste. Dietary fiber accounts for about 75–80% of the dry matter of pea seed coat and more than 80% is insoluble dietary fiber [1,2]. Relevant studies have shown that insoluble dietary fiber rich in various functional groups can control the sharp rise in postprandial blood glucose by adsorbing glucose, delaying glucose diffusion, and inhibiting the activity of starch digestive enzymes, thereby reducing the risk of type II diabetes [3,4,5,6]. However, the main components of insoluble dietary fiber (cellulose, hemicellulose, and lignin) interweave with each other in the cell walls to form a dense and stable network structure, which leads to low accessibility of fiber and limited exposure of active groups. This restriction limits the ability of the fiber to adsorb glucose and delay its diffusion, thus adversely affecting the hypoglycemic function of dietary fiber.

Ultrafine grinding technology is an effective way to achieve the physical modification of dietary fiber by using mechanical forces to destroy the intermolecular cohesion (aggregation of cellulose, hemicellulose, and lignin), resulting in micronization of dietary fiber [7,8]. Previous studies [9,10,11] have shown that ultrafine grinding can rapidly reduce the particle size to the cellular scale with a particle size below 50 μm. The specific surface area, porosity, surface energy, and exposure of functional groups of the particles are greatly increased with the grinding of dietary fiber particles, causing changes in the physicochemical, rheological, and biological properties of the dietary fiber [8,12,13]. These changes enhance the ability of dietary fiber to retain glucose, delay the diffusion of glucose, increase the number of fiber adsorption binding sites, and to improve its adsorption characteristics [14].

Previous studies [15,16,17] have shown that with the improvement of the microrefinement of dietary fiber, its intact cellular structure decreased and the fiber lobes increased, fiber component changed from insoluble to soluble, and the exposure of hydrophilic groups improved its water absorption and swelling ability, which ultimately affected the diffusion of glucose, starch and digestive enzymes in the digestive tract. The ultrafine grinding treatment at the cellular scale could increase the specific surface area and pore volume of dietary fiber, enhance the interception ability of glucose and digestive enzymes, and significantly increase the fiber adsorption binding sites, so that its adsorption capacity through Van der Waals force and hydrogen bonding could be significantly improved [18,19,20]. In addition, the adsorption effects of different components in dietary fiber are different, among which lignin could seriously hinder the contact between adsorbate and cellulose, and cause ineffective adsorption [21]. So how the adsorption characteristics of dietary fiber will be changed by the change in the position and content of each component after ultrafine grinding remains to be further studied.

Studies on the effects of ultrafine grinding treatment on dietary fiber have only provided qualitative descriptions [4,6,15,20]. Studies of dietary fiber describing quantitative correlation analysis and mechanisms linking particle size, microstructure properties, and glucose adsorption and diffusion have been rarely reported. In addition, the main roles and relative significance of microstructure parameters (particle size, fiber surface area, pore volume, cellulose crystallinity, surface element ratio) in the process of glucose adsorption and diffusion remain poorly understood, hindering directional and quantitative interventions of glucose adsorption and diffusion behaviors.

In this study, seven pea insoluble dietary fiber (PIDF) samples with different particle sizes were produced by ultrafine grinding, and the impact and mechanisms of ultrafine grinding on their microstructure features and the adsorption and diffusion behaviors of glucose in vitro were investigated. Furthermore, the quantitative relationships between particle size and microstructure parameters, as well as the contribution and relative significance of these parameters for adsorption and diffusion of glucose were analyzed. The results could lay a theoretical foundation for the controllable design of dietary fiber microstructure and its functional properties, which would be of great significance in controlling the fasting and postprandial blood glucose levels in the hyperglycemic population.

## 2. Materials and Methods

### 2.1. Materials

Fresh pea seed coats were obtained from Wenan County Lihe Grain and Oil Processing Company (Hebei, China). The seed coats were cleaned of contaminants, dried at 45 °C, and milled into coarse powder through a 40-mesh screen.

Glucose oxidase peroxidase kit was purchased from BioSino (Beijing, China). Alkaline proteinase and amyloglucosidase were purchased from Shanghai Yuanye (Shanghai, China). α-Amylase and glucose were purchased from Sigma (St. Louis, MO, USA). All other reagents used were of analytical grade.

### 2.2. Preparation of PIDF Samples with Different Particle Sizes

PIDF was extracted using an enzymatic method [19] with some modifications. Briefly, a known mass of pea seed coat powder was weighed and added into phosphate buffer solution (pH 9.1) at a solid-to-liquid ratio of 1:20 (w/v). Alkaline protease was added (600 U/g) and the mixture was heated at 60 °C for 30 min. After cooling, the pH was adjusted to 7.0, α-amylase (50 U/g) was added, and the mixture was heated at 90 °C for 30 min. After cooling to 60 °C, the pH was adjusted to 4.5, amyloglucosidase (300 U/g) was added, and the reaction was allowed to proceed at 60 °C for 30 min before the enzyme was destroyed by heating at 100 °C for 15 min. The reaction liquid was cooled and centrifuged at 4000 rpm for 10 min. The precipitate obtained was washed repeatedly until neutral, and then dried at 45 °C to obtain PIDF.

PIDF was coarsely milled using an RT-34 milling machine (Hongquan, Hong Kong, China) with the final sample passing through a 1.00 mm screen (sample denoted as BM0). An omnidirectional planetary ball mill (UBE-F2L; Changsha Tianchuang Powder Technology, Hunan, China) was used to produce samples of different particle sizes by mixing BM0 and ZrO_2_ balls in a volume ratio of 1:2 and milling for 30, 60, 100, 160, 240, and 400 min. The samples obtained by the respective milling times were denoted as BM30, BM60, BM100, BM160, BM240, and BM400.

### 2.3. Microstructure Characterization of PIDF

#### 2.3.1. Particle-Size Distribution

The particle size and distribution of PIDF samples were determined using a Mastersizer 2000 laser diffraction particle analyzer (Malvern, UK) with air as the dispersion medium and a measurement range of 0.01–3500 μm. Measurements were repeated three times for each sample. The particle-size distribution curves were used to determine the *D*_10_, *D*_50_, and *D*_90_ values, representing the 10th, 50th, and 90th percentiles of the total particles, respectively. The overall heterogeneity of the particle size for PIDF powders was evaluated as the span, which was calculated as (*D*_90_–*D*_10_)/*D*_50_ [22].

#### 2.3.2. Scanning Electron Microscopy (SEM) Images

Dried PIDF samples were uniformly adhered to a metal table with conductive tape, and the excess powder was gently blown off. The surface of samples was sputtered with Pt for 2 min using an E-1010 Iron Sputter (Hitachi, Tokyo, Japan). The surface morphologies of samples were observed with a Gemini300 thermal field-emission scanning electron microscope (Zeiss, Oberkochen, Germany) with a 5.0 kV accelerating voltage.

#### 2.3.3. Specific Surface Area (SSA) and Pore-Size Distribution

*SSA*s and pore-size distributions were determined according to the method of Liu et al., [23]. A known amount of dried PIDF sample was placed in a Micromeritics Tristar II specific surface and pore distribution instrument (Micromeritics, Norcross, GA, USA), and high-purity nitrogen gas was introduced for adsorption and desorption experiments. The measurement range of pore diameter was 2–200 nm. The BET model [24] was used to calculate the *SSA*, and the pore volume (*PV*) was estimated according to the Barrett-Joyner-Halenda (BJH) model [25]. Each sample was measured in duplicate.

#### 2.3.4. Cellulose Crystallinity (CrI)

A known amount of PIDF powder was compacted and placed on the instrument sample table and the X-ray diffraction spectrum was recorded using a D8 ADVANCE X-ray diffractometer (Bruker, Germany) with Cu-Kα radiation at 40 mA and 40 kV. The measurement mode was set in the 2θ range of 5–40° with a step size of 0.2°. Each sample was measured in duplicate. *CrI* was calculated according to the formula proposed by Segal et al., [26]:

(1) $$CrI\left(\%\right)=[\left(I_{002}-I_{\mathrm{am}}\right)/I_{002}]\times 100$$

where *I*_002_ is the diffraction peak intensity of crystal plane 002, and *I*_am_ is the intensity of the background peak at approximately 2*θ* = 18.0°.

#### 2.3.5. Surface Element Analysis

Surface elements were analyzed using an Escalab 250Xi photoelectron spectrometer (Thermo Fisher Scientific, Waltham, MA, USA) using monochromatic Al-Kα radiation at 1486.6 eV. The sample analysis area was 700 × 700 μm, and analysis depth was within 10 nm of the material surface. The full spectrum data and the narrow spectrum data of carbon (C1s) were obtained in the measurement. The surface element contents were calculated according to the area of the corresponding photoelectron peak of each element. The carbon peak spectrum (C1s) was divided by using Thermo Avantage software. The ratio of oxygen to carbon (*O/C*) was calculated by Equation (2) according to Ji et al., [27]. Each sample was measured in duplicate.

(2) $$O/C=I_{o}/2.85I_{c}$$

where *I_O_* is the normalized integrated area of the O1s peak, *I_C_* is the normalized integrated area of the C1s peak.

### 2.4. Determination of Glucose Adsorption Capacity

Glucose adsorption capacity (*GAC*) was measured in vitro according to the method of Zheng et al., [4] with some modifications. Each PIDF sample (0.5 g, recorded as *W*) was mixed with 25 mL (recorded as *V*) of glucose solution at different concentration (1, 5, 20, 50 mmol/L, 0.1 mol/L phosphate buffer solution adjusted pH to 6.9, recorded as *G*_1_). The mixture was placed in a shaker at 37 °C for 6 h, and then centrifuged at 4000 g for 15 min. The glucose content of the supernatant was measured by glucose assay kit and recorded as *G*_2_. Glucose solution without PIDF was regarded as a positive control. Each test was repeated three times and *GAC* (μmol/g) was calculated based on the difference of glucose concentration in external solution:

(3) $$GAC\;\left(\mu \mathrm{mol}/g\right)=\left(G_{2}-G_{1}\right)/W\times V$$

### 2.5. Measurement of Glucose Diffusion and Glucose Dialysis Retardation Index

Glucose diffusion and the glucose dialysis retardation index (*GDRI*) were determined in vitro according to the method of Qi et al., [6] with minor modifications. PIDF sample (0.2 g) was dispersed in 10 mL of glucose solution (100 mmol/L), and the mixture was dialyzed against 200 mL of distilled water at 37 °C using a dialysis membrane with a molecular weight cut-off of 12,000. After 10, 20, 40, 60, 90, 120, 180, and 300 min, the glucose content in the dialysate was measured using a glucose assay kit and was recorded as *G*_1_. A control test was carried out without the addition of PIDF sample to give *G*_2_. Each test was repeated three times. The GDRI was calculated according to Equation (4):

(4) $$GDRI\;\left(\%\right)=100-[\left(G_{1}/G_{2})\times 100\right]$$

The data for glucose content in dialysate (μmol, recorded as *y*) and time (min, recorded as *x*) were fitted with a parabolic equation [15,28]:

(5) $$y=ax^{2}+bx+c$$

where *a*, *b*, and *c* are coefficients. The equation to calculate the diffusion rate (*y*′) at any time is:

(6) $$y^{\prime }=2ax+b$$

When *x* is close to 0, *y*′ = the maximum diffusion velocity of glucose (*V*_max_) = *b*.

### 2.6. Statistical Analysis

Partial least squares (PLS) analysis was used to investigate the link between the *GAC* and microstructure properties of PIDF at different particle sizes. PLS analysis was performed in full cross validation with the “leave-one-out method” using the PLS toolbox of Matlab 2016b software (Mathworks, Natick, MA, USA).

All data were expressed as mean ± standard deviation. One-way analysis of variance (ANOVA) was used to compare means, significant difference analysis was based on Duncan’s multiple range test at the 95% level (*p* < 0.05), and correlation analysis was based on Pearson’s coefficient (*p* < 0.05, *p* < 0.01). Analyses were performed using SPSS Statistics version 23.0 (IBM, Armonk, NY, USA). Quantitative relationships were determined using Matlab 2016b software.

## 3. Results and Discussion

### 3.1. Microstructural Characterization of PIDF at Different Scales

#### 3.1.1. Particle-Size Distribution

**Figure 1:**
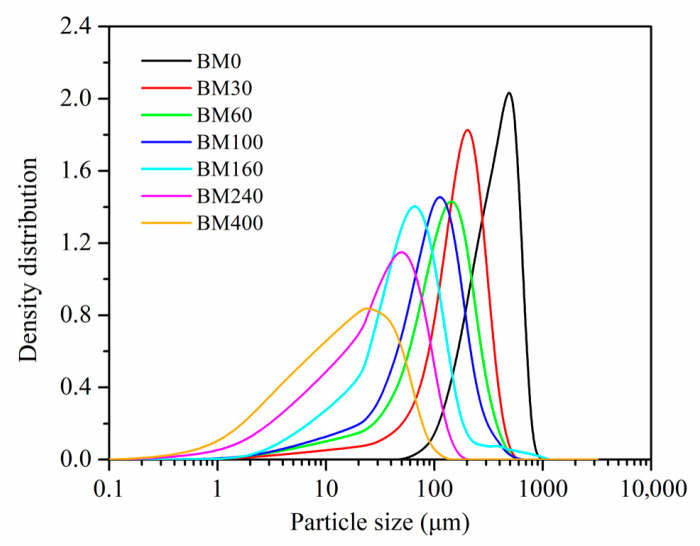
Particle size distribution curves of PIDF samples prepared using different grinding times.

The particle-size distributions and spans for PIDF samples produced with different grinding times are shown in Figure 1 and Table 1. The particle-size distribution curves of BM0 to BM400 samples gradually shifted to the left, indicating that the particle size successively decreased with grinding time. The particle sizes of BM0, BM30, BM60, BM100, and BM160 were tissue-scale crushed samples while they mostly distributed between 500 and 50 μm, whereas BM240 and BM400 were cellular-scale crushed samples with average particle size (*D*_50_) less than 50 μm [9,10]. With the extension of ultrafine grinding time, the *D*_50_ of the PIDF samples decreased significantly while the particle size span gradually increased from 1.14 ± 0.01 to 2.58 ± 0.21. The results showed that the prepared PIDF samples satisfied the classification requirements of powders of different scales and the data point requirements for establishing quantitative relationship models, which could lay a foundation for subsequent research on microstructure, glucose adsorption and diffusion intervention mechanism, and their quantitative relationship models.

#### 3.1.2. SEM Images

**Figure 2:**
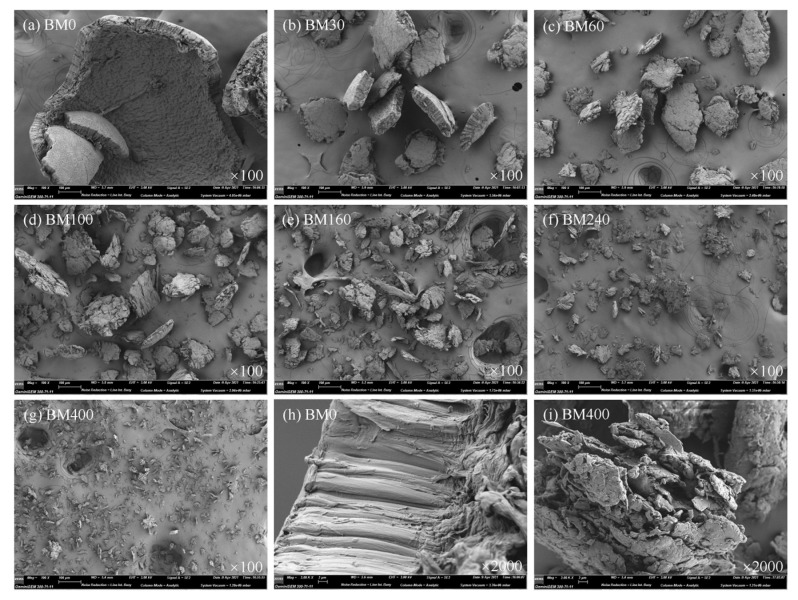
SEM images of PIDF samples at different scales.

The surface morphologies of PIDF samples at different scales are shown in Figure 2. At 100 times magnification (Figure 2a–g), the BM0 sample presented a relatively complete sheet-shell-like fiber structure with a compact and smooth surface, indicating that the main role of coarse grinding was to break and destroy the large fiber structure with little impact on its fiber bundle cell structure. With increased grinding time, the particle size of PIDF continued to decrease and the partly recognizable plant structures were gradually broken into small sheet-like structures as shown in BM30, BM60, and BM100 with rougher surfaces and more cracks. Furthermore, the tissue structures of BM160, BM240, and BM400 samples were basically completely destroyed by the prolonged crushing time, and the fibers appeared as short chains, strips and irregular particles. At 2000 times magnification (Figure 2h,i) comparison of BM0 with BM400 showed more loose scale structures and many deep pores of irregular shape and size in BM400. This structural complexity was likely caused by the strong extrusion and tearing action during the crushing process, which formed pores between PIDF fiber bundles. The abundant pore structure would greatly increase the accessibility to PIDF, and would be expected to improve its ability to adsorb glucose [29,30].

#### 3.1.3. Specific Surface Area and Pores

**Figure 3:**
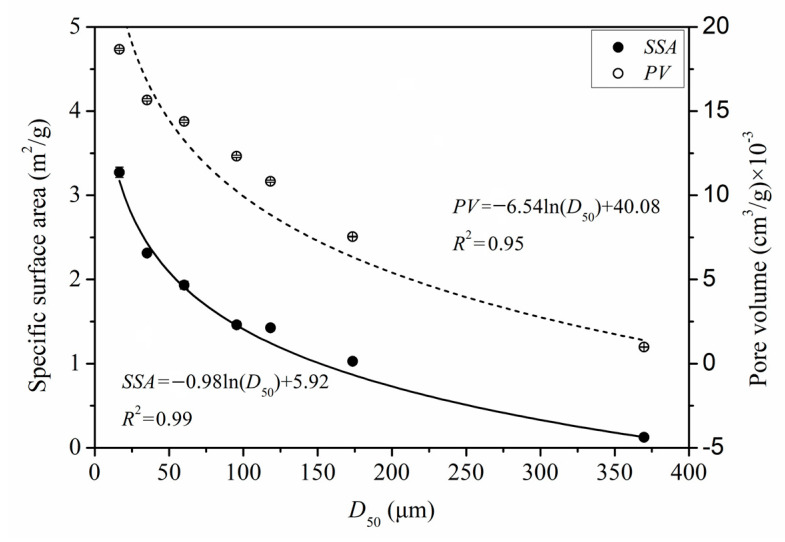
Quantitative relationships of *SSA*, *PV*, and *D*_50_ of PIDF.

The specific surface area (*SSA*) and pore volume (*PV*) data of PIDF at different scales are shown in Table S1. The *SSA* of the powders increased significantly with the increase in PIDF micronization. Previous studies have shown that the specific surface area of fiber can be divided into two parts: external and internal specific surface area. The former is inversely proportional to the particle size, while the latter mainly depends on the fiber porosity [11,31]. Compared with coarse grinding, ultrafine grinding significantly increased the *PV* as the particle size decreased by destroying the cell wall structure of fibers and exposing the internal pores [13,32]. Figure 3 shows the quantitative relationships between *SSA* and *D*_50_ (*SSA* = −0.98 × ln(*D*_50_) + 5.92, *R*^2^ = 0.99) and between *PV* and *D*_50_ (*PV* = −6.54 × ln(*D*_50_) + 40.08, *R*^2^ = 0.95). When PIDF particle size reached the cellular scale, the reduction in particle size significantly increased *SSA* and *PV*, and some researchers have reported similar conclusions [11,30].

**Figure 4:**
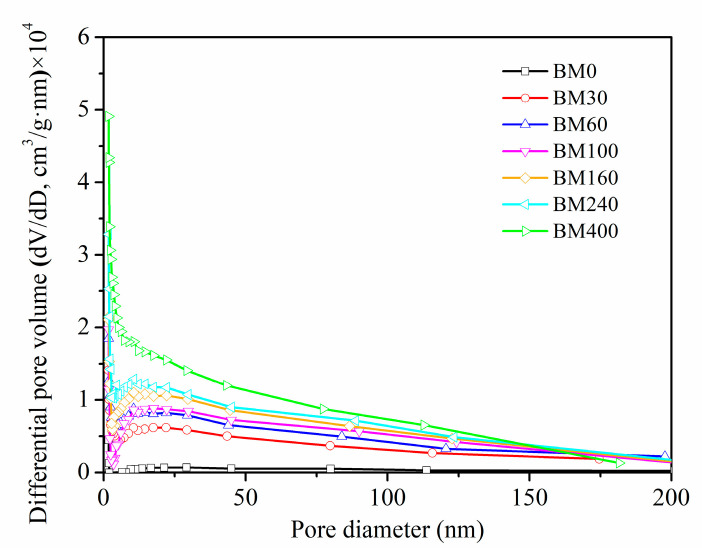
Pore size distribution of PIDF at different scales.

Figure 4 shows the pore size distributions of PIDF samples at different scales. The pore size distribution ranges (2–200 nm) of BM30-BM400 subjected to ultrafine grinding were wider than that of BM0 (2–50 nm) subjected to coarse grinding. In addition, the number of mesoporous (2–50 nm) and macroporous (>50 nm) structures exposed on the fiber surface increased significantly with the increase in micronization. SEM analysis of BM400 (Figure 2i) showed a scaly-porous structure with no sign of the plant tissue structure, with mesoporous and macroporous structures inside the fiber tissue exposed to the powder surface so that high-purity nitrogen could be adsorbed in them [23]. These structural changes help to improve the contact area between dietary fiber and glucose, and may improve adsorption ability and delay diffusion, thereby providing a means to stabilize postprandial blood glucose.

#### 3.1.4. Cellulose Crystallinity

**Figure 5:**
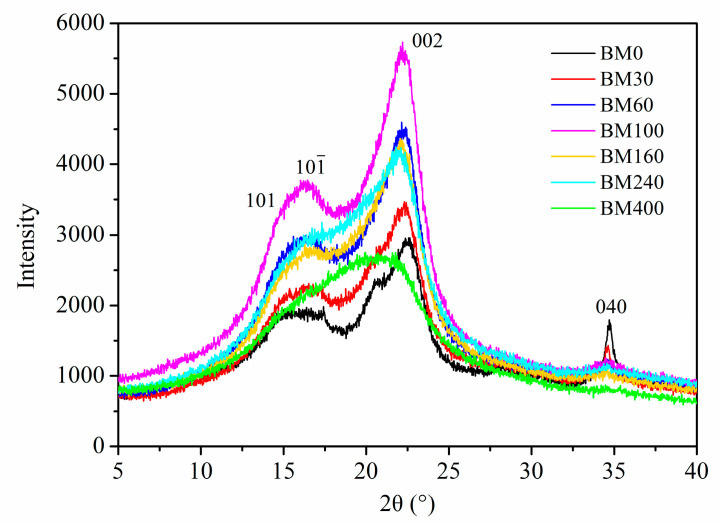
XRD patterns of PIDF samples at different scales.

Figure 5 shows the X-ray diffraction curves of PIDF at different scales. Three typical crystalline cellulose diffraction peaks were observed around 16°, 22°, and 35° in the tissue-scale PIDF samples (BM0, BM30, BM60, BM100, and BM160), which correspond to the crystal plane peaks of 101 and 101(—), 002, and 040 [33], exhibiting a cellulose type I crystal structure. For the cellular-scale PIDF samples, the crystalline cellulose diffraction peaks gradually decreased for BM240, while they disappeared for BM400, which showed a broad peak at approximately 2*θ* = 20°, indicating that the crystal structure of cellulose in the plant cell walls had been destroyed by ultrafine grinding at the cellular scale [34].

The *CrI* values of BM0, BM30, BM60, BM100, and BM160 (Table S1) were high and showed no significant difference (36.13–41.58%), indicating that the crystal structure was not seriously damaged. However, the *CrI* values of the cellular-scale samples (BM240, 29.58%; BM400, 12.33%) were significantly lower than those of the other lesser milled samples. This indicated that with the decrease in PIDF particle size, the hydrogen bonds between crystalline cellulose in the cell wall were destroyed, and the crystal structure became smaller, thus making the grain size smaller. When the grain size became sufficiently small, the crystal structure was severely damaged, leading to a significant decrease in crystallinity [22,35].

**Figure 6:**
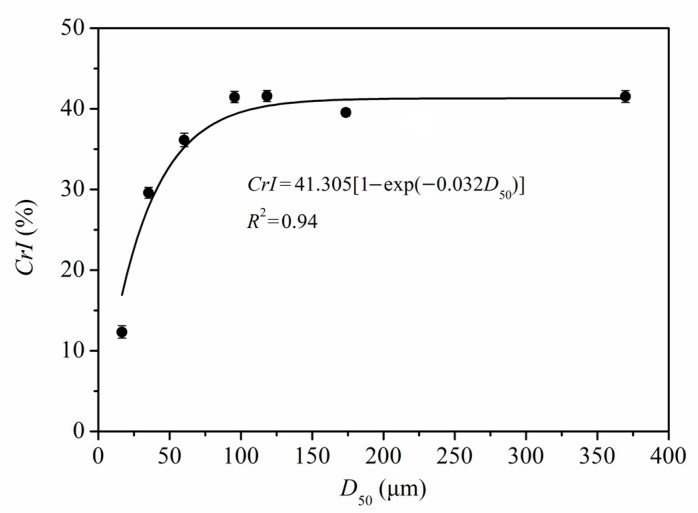
Quantitative relationship between *CrI* and *D*_50_ of PIDF.

Figure 6 shows the quantitative relationship between *CrI* and *D*_50_ of PIDF (*CrI =* 41.305 × [1 − exp(−0.032 × *D*_50_)], *R*^2^ = 0.94). According to the equation and fitted curve, the *CrI* of PIDF decreased with the decrease in particle size, but the decrease was exponential rather than linear. At the plant tissue scale, the *CrI* values did not decrease significantly even though the particle size decreased greatly (from 369.7 to 60.2 μm). However, when the PIDF particle size decreased to the cellular scale (35.2 and 16.6 μm), the *CrI* value decreased rapidly and the crystal structure was destroyed. Thus, it is predicted that the *CrI* value will be close to 0 and the crystallinity will be completely destroyed as the particle size of PIDF continues to decrease. Agarwal et al., [36] studied the influence of different ball milling times on the crystallinity of pure cellulose, and the results showed that the *CrI* of cellulose could be decreased from 78% to 0% by ball milling for 1 h. Ji et al., [12] studied the effects of different ball milling times on the particle size and crystallinity of rice straw. Their results showed that the corresponding crystallinity was 12.74% when the particle size was reduced to 13.67 μm by ball milling for 2 h, which basically conforms to the quantitative model observed in this study. The changes in fiber crystal structure caused by ultrafine grinding may have a beneficial effect on glucose adsorption properties.

#### 3.1.5. Surface Elemental Analysis

**Figure 7:**
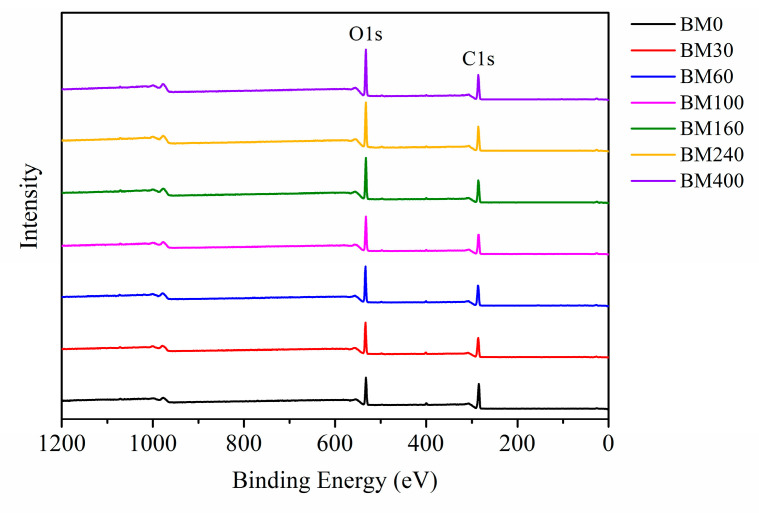
XPS spectra of PIDF at different scales.

XPS is a useful tool to evaluate the surface composition and chemical bond information of fiber materials [37]. Figure 7 shows the XPS full spectra of PIDF samples at different scales. Oxygen (O1s) and carbon (C1s) peaks were observed at 532 and 284 eV, respectively. The oxygen-to-carbon ratio (*O/C*) value of dietary fiber can reflect the relative content of oxygen-rich and carbon-rich substances on the sample surface [38]. Relevant studies [39,40] indicate that the theoretical *O/C* for cellulose/hemicellulose, lignin, and extractives are 0.83, 0.33, and 0.10, respectively. As shown in Table S2, the *O/C* of BM0 was 0.35, which is close to the *O/C* of pure lignin, indicating that the cellulose content on the surface of BM0 is low. This is because the coarse grinding did not cause much damage to the fiber bundle structure, and more cellulose was wrapped in the fiber bundle by lignin, and the surface may be covered by some wax and fat-soluble extractives [41,42]. The *O/C* values of the samples treated by ultrafine grinding (BM30 to BM400) increased significantly (from 0.43 to 0.56) as grinding time increased, which were between the *O/C* of cellulose/hemicellulose and lignin, indicating that the plant cell structure was destroyed, and cellulose and hemicellulose inside the cell wall were exposed to the sample surface [11,38].

**Figure 8:**
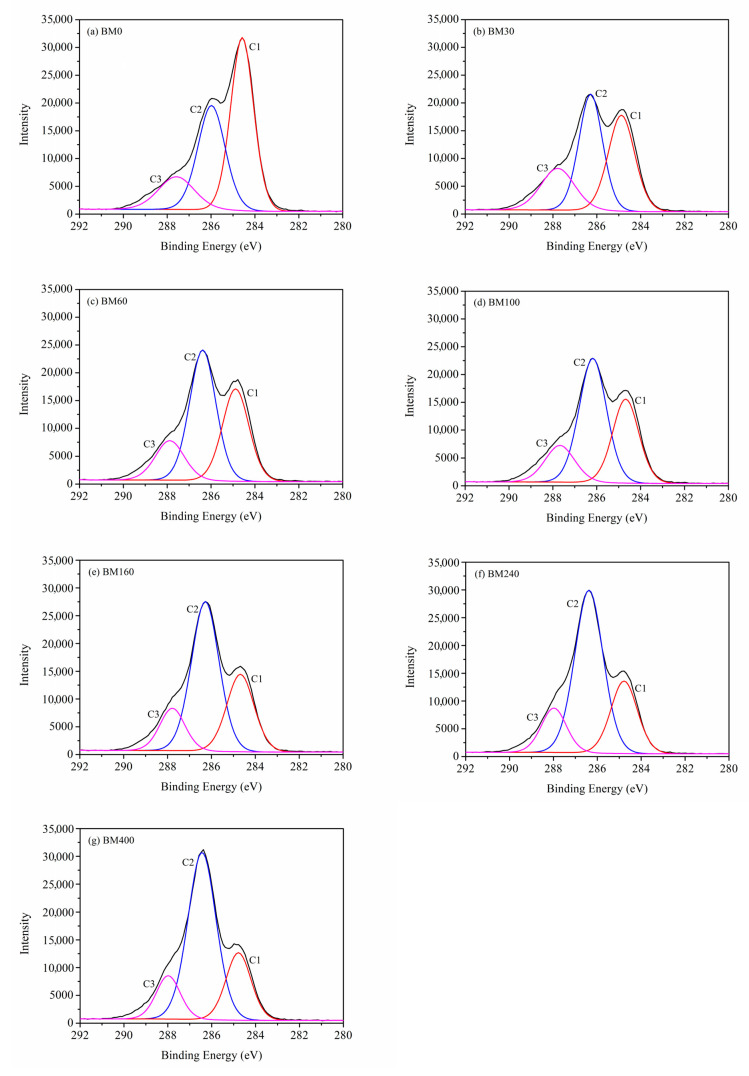
Deconvoluted peaks of C1s spectra for PIDF at different scales.

Figure 8 shows the deconvoluted peaks of C1s spectra for PIDF at different scales. C1s can be divided into C1, C2, and C3 peaks, which are located at 284.8, 286.3, and 287.8 eV, respectively [39,40]. C1 represents C-C/C-H, mainly derived from lignin and extractives; C2 represents C-O and is mainly derived from cellulose or hemicellulose; C3 corresponds to carbon atoms bonded to a carbonyl or two non-carbonyl oxygens (C=O/O–C–O) [38,43]. Table S2 shows that as the PIDF particle size decreased, the proportion of C1 decreased significantly, the proportion of C2 increased significantly, and the proportion of C3 did not change significantly. These results indicate that the cellulose and hemicellulose inside PIDF were more exposed to the powder surface and covered much of the lignin after ultrafine grinding treatment, which is consistent with the conclusion drawn from the change in surface *O/C*. The increase in surface cellulose/hemicellulose content may be beneficial for contact between PIDF and glucose, improve the glucose adsorption efficiency, and enhance its ability to delay glucose diffusion [11]. Lignin has been reported as a physical barrier that hinders the contact between adsorbate and cellulose, and the reduction in lignin relative content may have a positive effect on the adsorption of glucose and the ability to delay glucose diffusion [21,44].

**Figure 9:**
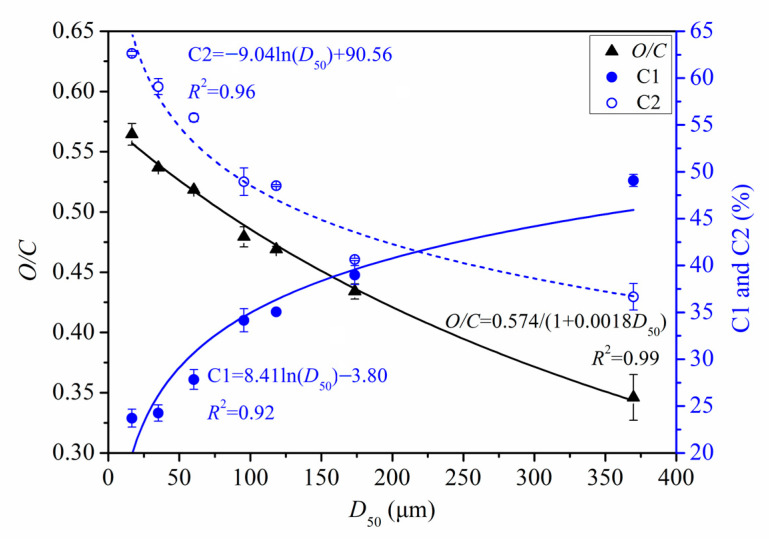
Quantitative relationship between surface elemental properties and *D*_50_ of PIDF.

Figure 9 shows the quantitative relationships between *D*_50_ and *O/C*, *D*_50_ and C1, and *D*_50_ and C2. *O/C* increased in a significant near-linear manner as *D*_50_ decreased. However, C1 and C2 showed logarithmic correlations with *D*_50_. As PIDF particle size decreased, the proportion of C1 decreased while C2 increased, with both trends becoming more pronounced at smaller particle size.

### 3.2. Glucose Adsorption Capacity

**Figure 10:**
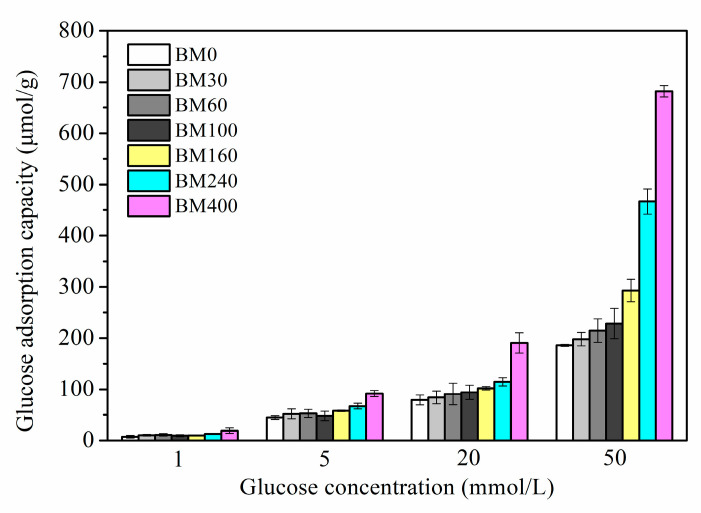
*GAC* determined at different glucose concentrations for PIDF at different scales.

Figure 10 shows the in vitro isothermal glucose adsorption results for PIDF samples. PIDF can effectively adsorb glucose at different glucose concentrations (1–50 mmol/L), and the glucose adsorption capacity (*GAC*) was proportional to the glucose concentration. *GAC* increased with the decrease in PIDF particle size. However, the improvement of *GAC* by ultrafine grinding at the tissue scale (BM30 to BM160) was limited. Although the reduction in particle size can improve the contact area between substrate and glucose, the biological resistance of samples is still at a high level due to the relative integrity of cell structure, resulting in low adsorption capacity after 6 h. In contrast, for the cellular-scale samples (BM240 and BM400), the *GAC* at different glucose concentrations was 1.5–2.5 times and 2.1–3.7 times higher than that of BM0, respectively. This result was reasoned in terms of the cellulose crystal structure in the cell walls of BM240 and BM400 being destroyed, which meant that the particle surface had high cellulose/hemicellulose content and low lignin/ extractives content. In addition, the mesopores and macropores inside the fiber were exposed to the particle surface and contributed to increases in *SSA* and *PV*. These changes resulted in the increase in the contact area between glucose and cellulose, and more glucose adsorption sites, which promoted the adsorption and binding of glucose on the fiber substrate. Chau et al., [45] found that the in vitro *GAC* of carrot insoluble dietary fiber after ball milling, jet milling, and high-pressure micronization increased by 205%, 254%, and 584%, respectively. In addition, when the glucose concentration was 1 mmol/L, PIDF could still adsorb a certain amount of glucose (7.28–19.02 μmol/g), indicating that PIDF could effectively keep the glucose concentration in the small intestine at a low level, thus preventing the occurrence of postprandial hyperglycemia.

### 3.3. Glucose Diffusion and Glucose Dialysis Retardation Index

**Figure 11:**
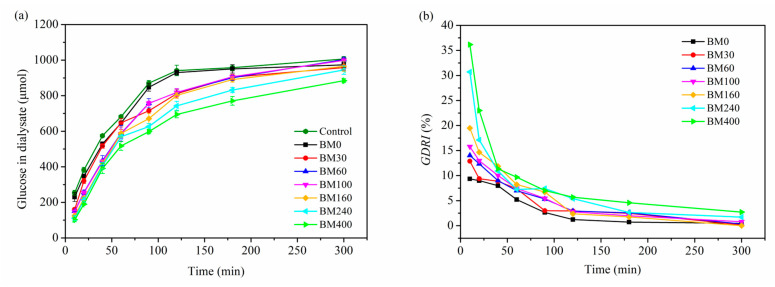
Effects of different PIDF samples on glucose diffusion (**a**) and *GDRI* (**b**).

Figure 11a shows the time dependence of glucose content in dialysate after treatment with PIDF samples. The glucose content in dialysate for treatments with BM0 to BM400 were 102.2–229.8 μmol at 10 min and 884.2–1003.1 μmol at 300 min. Compared with the control group, all PIDF samples showed inhibitory effects (0.72–59.68%) against glucose movement across the dialysis membrane into the external fluid, especially within the first 10 min. The glucose content in the dialysate for treatments with BM240 and BM400 were significantly lower than that of the other five treatments for the entire diffusion process. Data in Table 2 shows that the maximum diffusion velocity (*V*_max_) for treatments with BM0 to BM160 were 7.71–8.05 μmol/min (no significant difference), whereas the *V*_max_ for treatments with BM240 and BM400 decreased significantly to 7.00 and 6.58 μmol/min, respectively. These results indicated that the inhibition effect of PIDF on glucose diffusion in vitro was effectively enhanced by ultrafine grinding at the cellular scale.

The glucose dialysis retardation index (*GDRI*) has been used as a useful in vitro indicator of the effect of dietary fiber on gastrointestinal glucose absorption delay [46]. As shown in Figure 11b, all PIDF samples reached the maximum value of *GDRI* at 10 min; subsequently, the inhibitory ability of PIDF on glucose diffusion gradually weakened and became stable. The *GDRI*_max_ increased significantly with the decrease in particle size (Table 2). In the whole process of diffusion, BM400 had the strongest glucose diffusion inhibition ability (2.71–36.15%), which was about 1.4–5.8 times that of BM0 (0.47–9.35%). Similarly, some studies [15,20] have also shown that dietary fiber significantly inhibited glucose diffusion after ultrafine grinding at the cellular scale. Combined with the results for *GAC*, it is shown that BM400 had the strongest adsorption capacity for glucose, which indicates that adsorption is one of the main contributors to delaying glucose diffusion. Furthermore, relevant studies [4,6] have shown that the diffusion of glucose will be delayed by increased viscosity of the system, the physical barrier of fiber to glucose molecules, and the interception effect of fiber pore structure on the glucose. Considering that the insoluble dietary fiber contributes little to the viscosity of the system [28], it is apparent that the high dispersion of the ultrafine powder provides a large number of particles per unit of volume in the glucose-dietary fiber system [15,47]. These traits, together with the high *SSA* and porosity, enhance the binding of glucose, resulting in a greater physical barrier and delayed diffusion of glucose.

Based on the above findings, we conclude that cellular-scale ultrafine grinding of PIDF can enhance the in vitro abilities of glucose adsorption and delay diffusion, which means that PIDF has the potential to reduce glucose absorption in the intestine.

### 3.4. Correlation and Quantitative Relationship Analysis of Microstructure Parameters, GAC, and GDRI

#### 3.4.1. Pearson Correlation Analysis of Microstructure Parameters, GAC, and GDRI

Table 3 shows the correlation analysis of *GAC*, diffusion inhibition capacity, and the microstructure parameters of PIDF samples. There was a highly significant positive correlation between *GAC* and *SSA* (*p* < 0.01), significant positive correlations between *GAC* and *PV* and between *GAC* and *O/C* (*p* < 0.05), and a highly significant negative correlation between *GAC* and *CrI*. There were highly significant positive correlations between *GDRI*_max_ and *SSA* and between *GDRI*_max_ and *GAC*, significant positive correlations between *GDRI*_max_ and *PV* and between *GDRI*_max_ and *O/C*, significant negative correlation between *GDRI*_max_ and *D*_50_, and a highly significant negative correlation between *GDRI*_max_ and *CrI*. Therefore, we concluded that the changes of various microstructure parameters during ultrafine grinding have significant effects on glucose adsorption and diffusion, and the observed correlations can provide a theoretical basis to establish quantitative relationship models.

#### 3.4.2. Quantitative Relationships between the Microstructure Parameters and GAC

To describe the relative importance of each microstructure parameter to *GAC*, a PLS model was used to decouple and analyze the contribution of each parameter (*D*_50_, *SSA*, *PV*, *CrI*, *O/C*) to *GAC* at a glucose concentration of 50 mmol/L. In PLS analysis, variable importance in projection (*VIP*) is used to evaluate the weight of each microstructure parameter, where a larger *VIP* score indicates a more significant impact of the corresponding microstructure on *GAC*. Since the average of the squared *VIP* scores equals 1, the “greater-than-one rule” is generally used as a criterion for variable selection [48].

**Figure 12:**
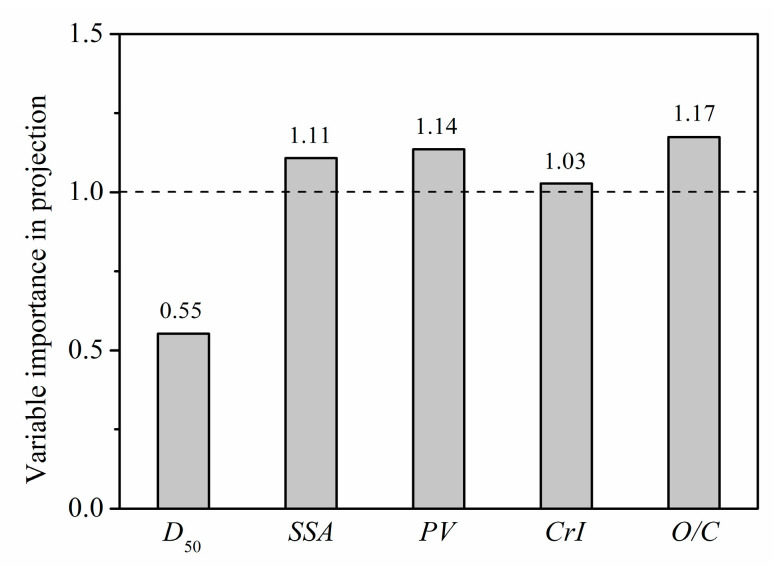
VIP scores of microstructure-property modifications for the PLS model.

Scores of *VIP* are shown in Figure 12. The microstructure parameters affecting *GAC* were sorted into the following order of importance: *O/C* > *PV* > *SSA* > *CrI* > D_50_. With the effect of *O/C* ranked highest, this result indicates that position transfer of cellulose, hemicellulose, and lignin, and the content change in each component on the fiber surface are key factors affecting the adsorption of glucose by PIDF. The *VIP* values of *PV*, *SSA*, and *CrI* were also greater than 1, indicating that the exposure of mesopores and macropores inside the fiber and the destruction of fiber crystal structure also had significant impacts on *GAC*. In comparison, the particle size (*D*_50_) had a minimum influence on adsorption, indicating that *GAC* mainly depends on the degree of damage to the internal structure of the fiber. This result suggests that reducing the particle size without destroying the tissue structure does not improve the adsorption rate. Only when the tissue structure of the fiber is destroyed and more glucose is brought into contact with the fiber can the adsorption capacity be effectively increased. These conclusions based on the influence of microstructural changes on *GAC* will provide some theoretical guidance for optimization of the grinding process.

**Figure 13:**
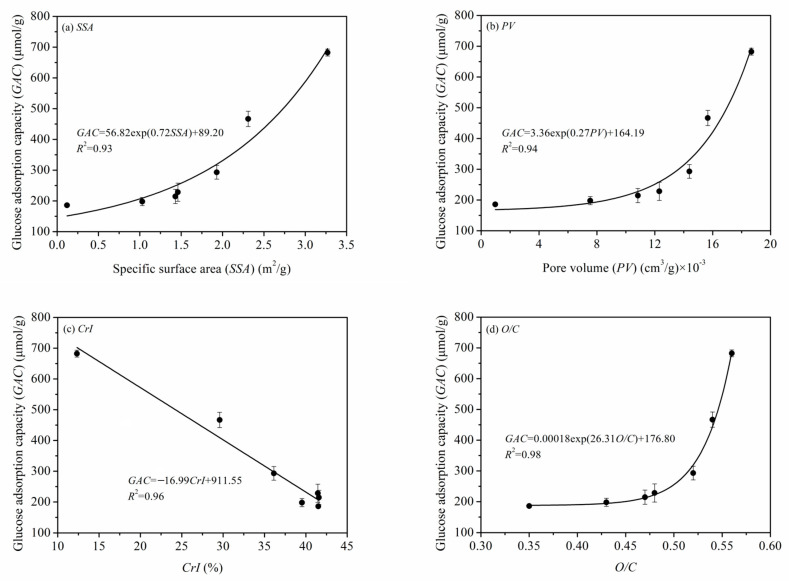
Quantitative relationships of SSA (**a**), PV (**b**), CrI (**c**), O/C (**d**) and GAC of PIDF.

The quantitative relationships between the microstructural properties of PIDF (*SSA*, *PV*, *CrI*, *O/C*) and *GAC* are shown in Figure 13. The relationships of *GAC* with *SSA*, *PV*, and *O/C* were exponential, that is, GAC increased slowly at first and then increased much more rapidly as the other parameter increased. The external and internal *SSA* are significantly increased when PIDF structure reaches the cellular scale after ultrafine grinding. At the same time, cellulose/hemicellulose displace lignin to become exposed on the surface of the particle, which significantly improves the contact area between PIDF and glucose and becomes the key factor in improving *GAC*. In contrast, *CrI* had a negative linear correlation with *GAC*. This result means that the crystallinity of cellulose is a negative factor that cannot be ignored because the integrity and compactness of the cellulose crystal structure may hinder the accessibility of glucose and affect the adsorption efficiency.

#### 3.4.3. Quantitative Relationship of D_50_, GAC, and GDRI_max_

**Figure 14:**
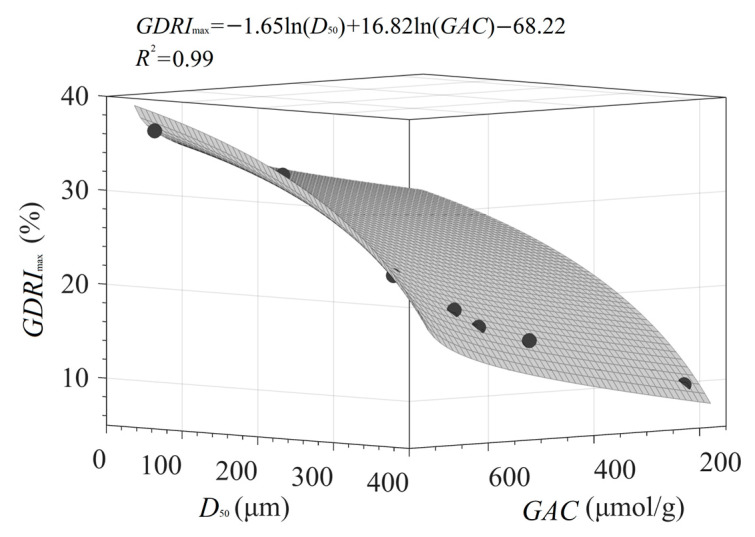
Quantitative relationship between *D*_50_, *GAC*, and *GDRI*_max_ of PIDF.

The data in Table 3 show that *GDRI*_max_ is significantly correlated with various microstructure parameters and *GAC*. The effects of *SSA*, *PV*, *CrI*, and *O/C* on *GDRI*_max_ can be attributed to the strong adsorption and binding of PIDF on glucose molecules, whereas the influence of particle size (*D*_50_) on *GDRI*_max_ is mainly reflected in the greater physical barrier caused by uniformly dense fiber particles per unit of volume. Therefore, taking *D*_50_ and *GAC* as independent variables and *GDRI*_max_ as the dependent variable, a binary nonlinear quantitative relationship [*GDRI*_max_ = −1.65 × ln(*D*_50_) + 16.82 × ln(*GAC*) − 68.22, *R*^2^ = 0.99] can be constructed (see Figure 14). The sign and magnitude of the coefficients in the equation [−1.65 for ln(*D*_50_) vs 16.82 for ln(*GAC*)] indicate that the retardation ability of fiber against glucose diffusion is negatively related to the particle size and positively related to *GAC*. However, the relative magnitude of the coefficients (16.82 > 1.65) also reflects the trends of the correlation data (Table 3), which suggested that the influence of glucose adsorption is significantly higher than that of fiber particle size. These results and models have considerable significance in efforts to achieve the quantitative and targeted intervention of dietary fiber structure for blood glucose control.

## 4. Conclusions

This study performed a thorough assessment of the effects of ultrafine grinding treatment on the microstructure of PIDF and its subsequent effects on glucose adsorption and diffusion in vitro. The results showed that the microstructure of PIDF changed to varying degrees in accordance with the decrease in particle size but became much more significant with ultrafine grinding at the cellular scale. With ultrafine grinding, the reticular structure of PIDF collapsed, the mesopores and macropores inside the fiber were exposed to the powder surface which lead to a significant increase in specific surface area and pore volume, a significant decrease in fiber crystallinity, an increase in the relative ratio of cellulose/hemicellulose and a decrease in the relative ratio of lignin/extractives on the surface, resulting in a significant increase in the contact area between glucose and cellulose, thus significantly improving the ability of PIDF to adsorb glucose and retard glucose diffusion. PLS analysis revealed the order of importance of microstructural changes on *GAC* as: *O/C* > *PV* > *SSA* > *CrI* > *D*_50_ with *GAC* showing positive exponential relationships with *SSA*, *PV*, and *O/C*, but a negative linear relationship with *CrI*. Furthermore, as a result of the adsorption and interception of glucose by fibers and the physical barrier effect of fiber density, the retardation ability of fiber particles to glucose diffusion was significantly improved with decreased particle size. A quantitative relationship of *D*_50_, *GAC* and *GDRI*_max_ was established as follows: *GDRI*_max_ = −1.65 ln(*D*_50_) + 16.82 ln(*GAC*)−68.22 (*R*^2^ = 0.99). This study demonstrated that ultrafine grinding is effective in improving the ability of insoluble dietary fiber to control blood glucose, which could provide new methodology and theoretical support for the development and application of PIDF as a promising low-calorie food ingredient.

## Figures and Tables

**Table 1:** Particle size and spans of PIDF samples at different scales.

Sample	*D*_10_ (μm)	*D*_50_ (μm)	*D*_90_ (μm)	Span
BM0	172.7 ± 0.2 ^g^	369.7 ± 2.9 ^g^	593.0 ± 0.8 ^g^	1.14 ± 0.01 ^a^
BM30	63.8 ± 0.5 ^f^	173.6 ± 0.9 ^f^	294.2 ± 1.6 ^f^	1.33 ± 0.00 ^a^
BM60	33.7 ± 0.7 ^e^	118.3 ± 0.2 ^e^	241.9 ± 0.7 ^e^	1.76 ± 0.01 ^b^
BM100	30.5 ± 0.8 ^d^	95.5 ± 0.4 ^d^	190.9 ± 2.4 ^d^	1.68 ± 0.01 ^b^
BM160	24.7 ± 0.0 ^c^	60.2 ± 0.1 ^c^	134.7 ± 1.1 ^c^	1.83 ± 0.01 ^b^
BM240	7.1 ± 0.1 ^b^	35.2 ± 0.1 ^b^	85.2 ± 1.9 ^b^	2.22 ± 0.05 ^c^
BM400	3.2 ± 0.5 ^a^	16.6 ± 0.3 ^a^	45.8 ± 3.2 ^a^	2.58 ± 0.21 ^d^

Values in same column with different letters are significantly different (*p* < 0.05).

**Table 2:** Glucose diffusion characteristics in the glucose-PIDF system.

Sample	Glucose Diffusion Equation	*R* ^2^	*V*_max_ (μmol/min)	*GDRI*_max_ (%)
Control	*y* = −0.0191*x*^2^ + 8.13*x* + 248.6	0.953	8.13	/
BM0	*y* = −0.0186*x*^2^ + 8.05*x* + 206.7	0.964	8.05	9.35
BM30	*y* = −0.0170*x*^2^ + 7.88*x* + 187.1	0.951	7.88	12.89
BM60	*y* = −0.0163*x*^2^ + 7.71*x* + 129.3	0.971	7.71	14.03
BM100	*y* = −0.0166*x*^2^ + 7.81*x* + 123.6	0.973	7.81	15.77
BM160	*y* = −0.0164*x*^2^ + 7.75*x* + 99.5	0.975	7.75	19.48
BM240	*y* = −0.0143*x*^2^ + 7.00*x* + 106.7	0.965	7.00	30.69
BM400	*y* = −0.0134*x*^2^ + 6.58*x* + 96.7	0.964	6.58	36.15

**Table 3:** Pearson correlation analysis of microstructure parameters with *GAC* and *GDRI*.

	*D*_50_	*SSA*	*PV*	*CrI*	*O/C*	*GAC*	*GDRI*_max_
*D*_50_	1						
*SSA*	−0.903 **	1					
*PV*	−0.977 **	0.967 **	1				
*CrI*	0.573	−0.865 *	−0.711	1			
*O/C*	−0.979 **	0.965 **	0.997 **	−0.711	1		
*GAC*	−0.642	0.902 **	0.774 *	−0.984 **	0.773 *	1	
*GDRI*_max_	−0.761 *	0.942 **	0.860 *	−0.927 **	0.866 *	0.974 **	1

* Significant correlation between parameters (*p* < 0.05). ** Highly significant correlation between parameters (*p* < 0.01).

## Data Availability

Data is contained within the article.

## Supplementary Materials

The following supporting information can be downloaded at: https://www.mdpi.com/article/10.3390/foods11182814/s1, Table S1: *SSA*, *PV*, and *CrI* values of PIDF samples at different scales, Table S2. Surface elemental characteristics of PIDF at different scales.

## References

[1] Martens L.G., Nilsen M.M., Provan F. (2017). Pea hull fibre: Novel and sustainable fibre with important health and functional properties. EC Nutr..

[2] Hashemi Z., Fouhse J., Im H., Chan C., Willing B. (2017). Dietary pea fiber supplementation improves glycemia and induces changes in the composition of gut microbiota, serum short chain fatty acid profile and expression of mucins in glucose intolerant rats. Nutrients.

[3] Jia M., Yu Q., Chen J., He Z., Chen Y., Xie J., Nie S., Xie M. (2020). Physical quality and in vitro starch digestibility of biscuits as affected by addition of soluble dietary fiber from defatted rice bran. Food Hydrocoll..

[4] Zheng Y., Wang Q., Huang J., Fang D., Zhuang W., Luo X., Zou X., Zheng B., Cao H. (2019). Hypoglycemic effect of dietary fibers from bamboo shoot shell: An in vitro and in vivo study. Food Chem. Toxicol..

[5] Arun K.B., Thomas S., Reshmitha T.R., Akhil G.C., Nisha P. (2017). Dietary fibre and phenolic-rich extracts from Musa paradisiaca inflorescence ameliorates type 2 diabetes and associated cardiovascular risks. J. Funct. Foods.

[6] Qi J., Li Y., Masamba K.G., Shoemaker C.F., Zhong F., Majeed H., Ma J. (2016). The effect of chemical treatment on the In vitro hypoglycemic properties of rice bran insoluble dietary fiber. Food Hydrocoll..

[7] Du B., Meenu M., Xu B. (2020). Insights into improvement of physiochemical and biological properties of dietary fibers from different sources via micron technology. Food Rev. Int..

[8] Ramachandraiah K., Chin K.B. (2016). Evaluation of ball-milling time on the physicochemical and antioxidant properties of persimmon by-products powder. Innov. Food Sci. Emerg..

[9] Barakat A., Monlau F., Solhy A., Carrere H. (2015). Mechanical dissociation and fragmentation of lignocellulosic biomass: Effect of initial moisture, biochemical and structural proprieties on energy requirement. Appl. Energy.

[10] Barakat A., Mayer-Laigle C., Solhy A., Arancon R.A.D., de Vries H., Luque R. (2014). Mechanical pretreatments of lignocellulosic biomass: Towards facile and environmentally sound technologies for biofuels production. RSC Adv..

[11] Ji G., Gao C., Xiao W., Han L. (2016). Mechanical fragmentation of corncob at different plant scales: Impact and mechanism on microstructure features and enzymatic hydrolysis. Bioresour. Technol..

[12] Ji G., Han L., Gao C., Xiao W., Zhang Y., Cao Y. (2017). Quantitative approaches for illustrating correlations among the mechanical fragmentation scales, crystallinity and enzymatic hydrolysis glucose yield of rice straw. Bioresour. Technol..

[13] Zhang H., Chen L., Lu M., Li J., Han L. (2016). A novel film-pore-surface diffusion model to explain the enhanced enzyme adsorption of corn stover pretreated by ultrafine grinding. Biotechnol. Biofuels.

[14] Protonotariou S., Mandala I., Rosell C.M. (2015). Jet milling effect on functionality, quality and in vitro digestibility of whole wheat flour and bread. Food Bioprocess Technol..

[15] Liu Y., Wang L., Liu F., Pan S. (2016). Effect of grinding methods on structural, physicochemical, and functional properties of insoluble dietary fiber from orange peel. Int. J. Polym. Sci..

[16] Zheng Y., Li Y. (2018). Physicochemical and functional properties of coconut (*Cocos nucifera* L.) cake dietary fibres: Effects of cellulase hydrolysis, acid treatment and particle size distribution. Food Chem..

[17] Srichamroen A., Chavasit V. (2011). In vitro retardation of glucose diffusion with gum extracted from malva nut seeds produced in Thailand. Food Chem..

[18] Chen H., Li J., Yao R., Yan S., Wang Q. (2020). Mechanism of lipid metabolism regulation by soluble dietary fibre from micronized and non-micronized powders of lotus root nodes as revealed by their adsorption and activity inhibition of pancreatic lipase. Food Chem..

[19] Zhu F., Du B., Li R., Li J. (2014). Effect of micronization technology on physicochemical and antioxidant properties of dietary fiber from buckwheat hulls. Biocatal. Agric. Biotechnol..

[20] Chen J., Gao D., Yang L., Gao Y. (2013). Effect of microfluidization process on the functional properties of insoluble dietary fiber. Food Res. Int..

[21] Siqueira G., Arantes V., Saddler J.N., Ferraz A., Milagres A.M.F. (2017). Limitation of cellulose accessibility and unproductive binding of cellulases by pretreated sugarcane bagasse lignin. Biotechnol. Biofuels.

[22] Silva G.G.D., Couturier M., Berrin J., Buléon A., Rouau X. (2012). Effects of grinding processes on enzymatic degradation of wheat straw. Bioresour. Technol..

[23] Liu T.Y., Ma Y., Yu S.F., Shi J., Xue S. (2011). The effect of ball milling treatment on structure and porosity of maize starch granule. Innov. Food Sci. Emerg..

[24] Brunauer S., Emmett P.H., Teller E. (1938). Adsorption of gases in multimolecular layers. J. Am. Chem. Soc..

[25] Barrett E.P., Joyner L.G., Halenda P.P. (1951). The determination of pore volume and area distributions in porous substances. I. computations from nitrogen isotherms. J. Am. Chem. Soc..

[26] Segal L., Creely J.J., Martin A.E., Conrad C.M. (1959). An empirical method for estimating the degree of crystallinity of native cellulose using the X-Ray diffractometer. Text. Res. J..

[27] Ji G., Xiao W., Gao C., Cao Y., Zhang Y., Han L. (2018). Mechanical fragmentation of wheat and rice straw at different scales: Energy requirement in relation to microstructure properties and enzymatic hydrolysis. Energy Convers. Manag..

[28] Ou S., Kwok K., Li Y., Fu L. (2001). In vitro study of possible role of dietary fiber in lowering postprandial serum glucose. J. Agric. Food Chem..

[29] Arantes V., Saddler J.N. (2011). Cellulose accessibility limits the effectiveness of minimum cellulase loading on the efficient hydrolysis of pretreated lignocellulosic substrates. Biotechnol. Biofuels.

[30] Zhang H., Chen L., Li J., Lu M., Han L. (2017). Quantitative characterization of enzyme adsorption and hydrolytic performance for ultrafine grinding pretreated corn stover. Bioresour. Technol..

[31] Li H., Ye C., Liu K., Gu H., Du W., Bao J. (2015). Analysis of particle size reduction on overall surface area and enzymatic hydrolysis yield of corn stover. Bioprocess Biosyst. Eng..

[32] Tumuluru J.S., Tabil L.G., Song Y., Iroba K.L., Meda V. (2014). Grinding energy and physical properties of chopped and hammer-milled barley, wheat, oat, and canola straws. Biomass Bioenergy.

[33] Park S., Baker J.O., Himmel M.E., Parilla P.A., Johnson D.K. (2010). Cellulose crystallinity index: Measurement techniques and their impact on interpreting cellulase performance. Biotechnol. Biofuels Bioprod..

[34] Peng H., Li H., Luo H., Xu J. (2013). A novel combined pretreatment of ball milling and microwave irradiation for enhancing enzymatic hydrolysis of microcrystalline cellulose. Bioresour. Technol..

[35] Yang Y., Ji G., Xiao W., Han L. (2014). Changes to the physicochemical characteristics of wheat straw by mechanical ultrafine grinding. Cellulose.

[36] Agarwal U.P., Zhu J.Y., Ralph S.A. (2013). Enzymatic hydrolysis of loblolly pine: Effects of cellulose crystallinity and delignification. Holzforschung.

[37] Sain M., Panthapulakkal S. (2006). Bioprocess preparation of wheat straw fibers and their characterization. Ind. Crop. Prod..

[38] Chundawat S.P.S., Venkatesh B., Dale B.E. (2007). Effect of particle size based separation of milled corn stover on AFEX pretreatment and enzymatic digestibility. Biotechnol. Bioeng..

[39] Dorris G.M., Gray D.G. (1978). The surface analysis of paper and wood fibers by ESCA II. Cell. Chem. Technol..

[40] Dorris G.M., Gray D.G. (1978). The surface analysis of paper and wood fibers by ESCA I. Cell. Chem. Technol..

[41] Rjiba N., Nardin M., Dréan J.Y., Frydrych R. (2007). A study of the surface properties of cotton fibers by inverse gas chromatography. J. Colloid Interface Sci..

[42] Inari G.N., Pétrissans M., Dumarcay S., Lambert J., Ehrhardt J.J., Šernek M., Gérardin P. (2011). Limitation of XPS for analysis of wood species containing high amounts of lipophilic extractives. Wood Sci. Technol..

[43] Hua X., Kaliaguine S., Kokta B.V., Adnot A. (1993). Surface analysis of explosion pulps by ESCA Part 1. Carbon (1s) spectra and oxygen-to-carbon ratios. Wood Sci. Technol..

[44] Djajadi D.T., Jensen M.M., Oliveira M., Jensen A., Thygesen L.G., Pinelo M., Glasius M., Jørgensen H., Meyer A.S. (2018). Lignin from hydrothermally pretreated grass biomass retards enzymatic cellulose degradation by acting as a physical barrier rather than by inducing nonproductive adsorption of enzymes. Biotechnol. Biofuels.

[45] Chau C., Wang Y., Wen Y. (2007). Different micronization methods significantly improve the functionality of carrot insoluble fibre. Food Chem..

[46] López G., Ros G., Rincón F., Periago M.J., Martínez M.C., Ortuño J. (1996). Relationship between physical and hydration properties of soluble and insoluble fiber of artichoke. J. Agric. Food Chem..

[47] Zhao X., Yang Z., Gai G., Yang Y. (2009). Effect of superfine grinding on properties of ginger powder. J. Food Eng..

[48] Farrés M., Platikanov S., Tsakovski S., Tauler R. (2015). Comparison of the variable importance in projection (VIP) and of the selectivity ratio (SR) methods for variable selection and interpretation. J. Chemom..

