# Supplementary material for: Qualitative and Quantitative Correlation of Microstructural Properties and In Vitro Glucose Adsorption and Diffusion Behaviors of Pea Insoluble Dietary Fiber Induced by Ultrafine Grinding
Source: Foods. 2022 Sep 13;11(18):2814. doi: 10.3390/foods11182814 (PMC9497999; doi:10.3390/foods11182814)
Supplement: Supplementary file 1 [file foods-11-02814-s001.zip › foods-1897830-supplementary.pdf]

Qualitative and Quantitative Correlation of Microstructural Properties and in Vitro Glucose Adsorption and Diffusion Behaviors of Pea Insoluble Dietary Fiber Induced by Ultrafine Grinding

Supplementary Materials

Lingyi Li [1, 2], Jianfu Liu [1,2,*], Yang Zhang [1,2], Qian Wang [1,2] and Jinrong Wang [1,2]

[1] Tianjin Key Laboratory of Food and Biotechnology, School of Biotechnology and Food Science, Tianjin University of Commerce, No. 409 Guangrong Road, Beichen District, Tianjin 300134, China

[2] Tianjin International Joint Research and Development Center, No. 409 Guangrong Road, Beichen District, Tianjin 300134, China

* Corresponding Author: ljf@tjcu.edu.cn

Table S1. *SSA*, *PV*, and *CrI* values of PIDF samples at different scales.

Sample	*SSA* (m^2/g)	*PV* (cm^3/kg)	*CrI* (%)
BM0	0.12 ± 0.00^a	0.98 ± 0.01^a	41.51 ± 0.73^d
BM30	1.03 ± 0.04^b	7.54 ± 0.03^b	39.53 ± 0.46^d
BM60	1.43 ± 0.04^c	10.83 ± 0.06^c	41.58 ± 0.68^d
BM100	1.46 ± 0.04^c	12.31 ± 0.06^d	41.45 ± 0.69^d
BM160	1.93 ± 0.05^d	14.39 ± 0.07^e	36.13 ± 0.84^c
BM240	2.31 ± 0.03^e	15.66 ± 0.07^f	29.58 ± 0.68^b
BM400	3.27 ± 0.06^f	18.67 ± 0.06^g	12.33 ± 0.76^a

Values in same column with different letters are significantly different ($p < 0.05$).

Table S2. Surface elemental characteristics of PIDF at different scales.

Sample	*O*/*C*	C1 (%)	C2 (%)	C3 (%)
BM0	0.35 ± 0.02^a	49.08 ± 0.65^e	37.02 ± 1.41^a	13.91 ± 0.77^a
BM30	0.43 ± 0.01^b	38.98 ± 1.02^d	41.01 ± 0.12^b	20.02 ± 0.90^b
BM60	0.47 ± 0.00^c	35.05 ± 0.12^c	48.89 ± 0.11^c	16.07 ± 0.23^a
BM100	0.48 ± 0.01^c	34.15 ± 1.23^c	49.30 ± 1.47^c	16.55 ± 0.24^a
BM160	0.52 ± 0.00^d	27.83 ± 1.05^b	56.14 ± 0.43^d	16.03 ± 1.48^a
BM240	0.54 ± 0.01^{de}	24.26 ± 0.88^a	59.45 ± 0.85^e	16.30 ± 1.73^a
BM400	0.56 ± 0.01^{ef}	23.71 ± 0.95^a	62.99 ± 0.23^f	13.30 ± 0.72^a

Values in same column with different letters are significantly different ($p < 0.05$).
